# Disrupted circadian rhythms and opioid‐mediated adverse effects: Bidirectional relationship and putative mechanisms

**DOI:** 10.1111/jne.70065

**Published:** 2025-07-06

**Authors:** Nasrin Mehranfard, Maedeh Ghasemi, Ehsan Saboory

**Affiliations:** ^1^ Nanokadeh Darooee Samen Private Joint Stock Company Urmia Iran; ^2^ Department of Physiology, School of Medicine Isfahan University of Medical Sciences Isfahan Iran; ^3^ Department of Physiology, School of Medicine University of Kyrenia Kyrenia North Cyprus

**Keywords:** circadian rhythms, neuronal excitability, opioids, oxidative stress, stress

## Abstract

Recent studies have shown a link between disrupted circadian rhythms and the development of chronic opioid‐induced negative effects. Both animal and human studies show a significant bidirectional relationship between the circadian system and opioid effects. Opioids can perturb circadian rhythms, and perturbation of the circadian rhythms can aggravate opioid‐mediated adverse effects. These bidirectional interactions may attenuate the outcomes of long‐term opioid therapy when not considered. A better understanding of the potential mechanisms underlying these interactions may be essential for more effective management of opioid‐induced adverse effects. This review highlights the association between circadian rhythms and opioid‐induced hyperalgesia, dependence, and withdrawal, and the possible role of the dopaminergic, serotoninergic, and noradrenergic systems, redox state, and stress in this association. We also highlight the existence of an interaction between other rhythmic biological processes, including the sleep–wake cycle as well as melatonin and glucocorticoid rhythms on the circadian and opioid systems and their possible effects on opioid‐related negative effects.

## 1 INTRODUCTION

The use of opioids, both in therapeutic purposes and substance misuse contexts, has been shown to disrupt the timing and integrity of the circadian system.^1^ Moreover, opioid withdrawal has been associated with disrupted circadian rhythms, which in turn lead to negative mental states and increased cravings.^2^ On the other hand, a disruption in the integrity of the circadian system can alter the efficacy and impacts of opioids.^3^ Despite this close relationship between the systems, the complex relationship between the biological clock and opioid‐induced adverse effects (complications of opioid therapy) is far from being understood. Thus, understanding the mechanisms underlying circadian rhythm disruptions associated with opioid use might guide us to develop therapies that can improve the effectiveness of opioid treatment for more effective management of opioid‐induced adverse effects and to develop therapies and interventions for opiate addicts. Moreover, given that the circadian and opioid systems are influenced by other rhythmic biological processes, including the sleep–wake cycle,^4^ corticosterone and melatonin rhythms, hence, this review also highlights the association between circadian rhythm, the opioid system, and these rhythmic biological processes and their possible role in diminishing opioid‐induced adverse effects.

We performed literature searches in PubMed, Scopus, ScienceDirect, and Google Scholar. Our search strategy covered terms related to “circadian rhythms and opioids,” “circadian rhythms and neurotransmitters,” “circadian rhythms and sleep/wake,” “circadian rhythms and melatonin,” “circadian rhythms and oxidative stress,” “circadian rhythms and stress,” “circadian rhythms and sex differences,” “circadian rhythms and genetic,” and “circadian rhythms and age,” Furthermore, terms related to opioids and neurotransmitters, sleep/wake, melatonin, oxidative stress, stress, sex differences, genetic, and age were also searched.

## 2 THE ENDOGENOUS OPIOID SYSTEM

The endogenous opioid system includes widely scattered neurons that produce opioid peptides. Three major types of endogenous opioid peptides have been identified in the central nervous system (CNS) and peripheral tissues: enkephalins, endorphins, and dynorphins. These peptides are derived from three precursors (i.e., proopiomelanocortin, proenkephalin, and prodynorphin).^5^

Opioid peptides exert their effects by acting on all three of the opioid receptor types, μ, δ, and κ, although with different affinities.^5^ These opioid receptors are members of the superfamily of G protein‐coupled receptors (GPCRs), and their activation suppresses neuronal firing and the cAMP signaling pathway.^6^, ^7^, ^8^, ^9^ The different types of opioid receptors bind to their respective endogenous ligands: μ‐opioid receptors bind to beta‐endorphin and endomorphin 1 and 2, κ‐opioid receptors bind to dynorphin A and B, and δ‐opioid receptors bind to enkephalins.^10^

The endogenous opioid system plays an important role in regulating a wide range of physiologic functions, including nociceptive transmission and pain perception, immune‐related responses, stress resilience, euphoria, digestive tract motility, food intake control, and cardioprotection.^11^ Different types of opioid receptors, related endogenous ligands, and their actions are summarized in Table 1.

**TABLE 1:** Different types of opioid receptors, related endogenous ligands, and their actions.

Opioid receptor	Endogenous ligands	Actions
μ‐opioid receptors	Beta‐endorphin, endomorphin 1 and 2	For analgesia and dependence
κ‐opioid receptors	Dynorphin A and B	For analgesia and dysphoria
δ‐opioid receptors	Enkephalins (met‐enkephalin and leu‐enkephalin)	For analgesia, hedonia, and mood

## 3 SHORT‐TERM AND LONG‐TERM EFFECTS OF OPIOIDS

Short‐term opioid‐based treatments can help patients alleviate pain. However, opioid chronic use can cause a reduction in the analgesic impact (tolerance) and induce hyperalgesia, which in turn can increase opioid use and the risk of addiction.^12^, ^13^ Human studies have reported an increase in comorbid psychiatric disorders (i.e., the coexistence of two or more psychiatric disorders) and premature death after chronic opioid use.^14^ Also, attention‐deficit hyperactivity disorder (ADHD) symptoms were found in 19.4% of heroin‐dependent patients.^15^ Additionally, the pain‐relieving effect of opioids is mostly associated with euphoric and rewarding impacts, which could cause misuse. This, in turn, leads to both physical and psychological dependence^16^ and may result in opioid use disorders.

Opioid use disorders are chronic brain diseases, which result from long‐term molecular and cellular neuroadaptations in the CNS,^17^, ^18^, ^19^, ^20^, ^21^, ^22^, ^23^, ^24^ that develop following repeated opioid use and withdrawal episodes. These neuroadaptations, in turn, result in a negative affect (i.e., negative mood states) characterized by increased anxiety, social deficits, dysphoria, sleep disorders, chronic irritability, emotional and/or physical pain, and reduced motivation for natural rewards. The development of this negative affect following long‐term opioid use is one of the main causes of relapse and further restricts the clinical utility of these medications. Thus, a better understanding of the neural mechanisms involved in opioid use disorders is essential to counteract the most detrimental and devastating effects of chronic opioid therapy.

## 4 THE CIRCADIAN CLOCK

Most organisms exhibit daily behavioral and/or physiological rhythms allowing them to adapt to cyclic environmental changes and react to different external cues called zeitgebers, such as light, hormonal signals, and food intake. These rhythms are known as circadian (i.e., a period of ~24 h) and are driven by an independent, internal timekeeping system termed the circadian clock.^25^, ^26^ The circadian molecular clock machinery is found in all tissues of the body.^25^ The suprachiasmatic nucleus (SCN) of the hypothalamus is the master circadian clock in mammals.^25^ Multiple accessory extra‐SCN oscillators have also been identified in the brain and peripheral tissues that are synchronized by the SCN or can be regulated independently. Various environmental cues or zeitgebers reset the clock system via the SCN or directly through acting on the tissue clock.^27^

**FIGURE 1:**
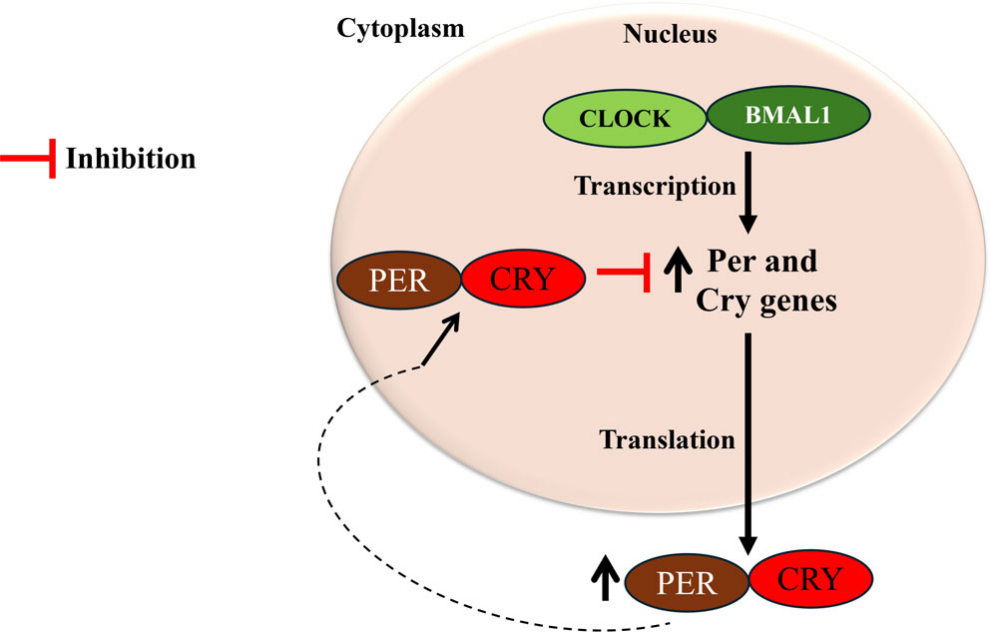
The circadian clock and transcriptional‐translational feedback loop. The heterodimer CLOCK/BMAL1 activates the expression of circadian clock genes (e.g., period (Per) and cryptochrome (Cry)). PER and CRY proteins bind together, and this complex then negatively regulates the activity of CLOCK‐BMAL1.

A network of transcriptional–translational feedback loops is located at the basic core of the circadian clock, and its principal components are recognized as clock genes. These genes form the molecular clock, and their protein products are essential for circadian rhythm generation within individual cells.^28^ The primary feedback loop consists of three transcription factors, including neuronal PAS domain protein 2 (NPAS2), circadian locomotor output cycles kaput (CLOCK), and brain and muscle ARNT‐like protein 1 (BMAL1). CLOCK:BMAL1 heterodimers stimulate the transcription of period (Per1, Per2, and Per3) and cryptochrome (Cry1 and Cry2) genes.^28^ PER:CRY complexes translocate back to the nucleus to inhibit their own transcription by acting on the CLOCK:BMAL1 heterodimers in a negative feedback.^25^, ^28^ The transcriptional–translational feedback loop is shown in Figure 1.

## 5 CIRCADIAN RHYTHMS IN THE ENDOGENOUS OPIOID SYSTEM

Both the basal pain sensitivity and analgesic effect of morphine show notable 24‐h variations.^29^, ^30^, ^31^ For example, in mice, the analgesic effect of morphine showed a diurnal rhythm, with an augmented analgesic impact in mice exposed to dark conditions.^31^ Also, a retrospective review of patients with cancer pain treated with morphine demonstrated a variation of morphine demand over a 24‐h period for the patients, with less opioid demand during the night.^32^ These variations in morphine analgesia are mostly attributed to the circadian fluctuations of the endogenous opioid system.^33^, ^34^, ^35^, ^36^

Earlier studies have demonstrated that opioid peptides are secreted into the extracellular milieu under a circadian rhythm control.^37^, ^38^ Several studies have revealed that the endogenous opioid system (i.e., opioid peptides and receptors) shows a circadian rhythm such that the tissue content of opioid peptides and the number of opioid receptors are highest during the night (24:00 h) and tend to be lowest in the early mornings (05:00 h).^34^, ^37^ Asai et al. (2007) also reported that the tissue content of met‐enkephalin, leu‐enkephalin, and synenkephalin (a non‐opioid peptide derived from proenkephalin) was enhanced during the dark phase (21:00–01:00 h) period in the hippocampus and hypothalamus in rats,^38^ consistent with an enhanced number of opioid receptors.^39^

Opioid receptors are highly expressed in various brain areas, including those regulating circadian rhythms such as, the locus coeruleus (LC),^40^ the dorsal raphe nucleus, as well as sleep/wake and circadian centers in the hypothalamus.^2^ Opioid drugs serve their impacts primarily by acting at the μ‐opioid receptor, but they also act at the κ‐ and δ‐opioid receptors to a lesser extent.^41^ The μ‐opioid receptor is thought to be responsible for opioid analgesia and dependence. In this regard, the desensitization of μ‐opioid receptor following chronic opioid use was accompanied by opioid‐induced hyperalgesia and reduced effectiveness of opioid therapy owing to the development of analgesic tolerance.^13^, ^42^ Other opioid receptors, including the κ‐opioid receptors, are linked to opioid‐induced dysphoria^7^ and the δ‐opioid receptors are implicated in the hedonic properties of opioids, as well as their effects on mood.^7^ The κ‐ and δ‐opioid receptors are also involved in the analgesic effect of opioids.^10^ Additionally, the δ‐opioid receptors, but not the μ‐ or κ‐opioid receptors, were found to produce non‐photic phase shifts in hamster activity rhythms.^43^, ^44^, ^45^ Moreover, δ‐opioid receptor‐labeling surrounding cells were found in the SCN in hamsters, suggesting that the δ‐opioid receptors are expressed at presynaptic terminals.^43^ Circadian rhythms in the endogenous opioid system are summarized in Table 2.

**TABLE 2:** Summary of circadian rhythms in the endogenous opioid system.

Endogenous opioid system components	Circadian rhythms
Endogenous opioid peptides (enkephalins, endorphins, and dynorphins)	Peak tissue level during the night (24:00–01:00 h). Lowest level in the early mornings (05:00 h).
Opioid receptors (μ, δ, and κ receptors)	Highest during the night (24:00 h) and lowest in the early mornings (05:00 h). Highly expressed in sleep/circadian centers (in locus coeruleus, dorsal raphe nucleus, and hypothalamus). Opioid receptor activation inhibits cAMP production, which displays circadian variations. Rhythmic variations in the expression of μ‐opioid receptor have been shown in mouse brainstem. δ‐receptors: Produce non‐photic phase shifts in hamster activity rhythms. Likely found at presynaptic terminals in SCN hamster.

## 6 INTERACTION BETWEEN THE CIRCADIAN CLOCK AND OPIOIDS

### 6.1 Effect of the circadian clock on the opioid system

Per2 (Brdm1) mutant mice showed a decrease in morphine tolerance and withdrawal signs compared with wild‐type control mice.^46^ In addition, mice treated with ribozyme (to interfere with mPer1 gene expression in the CNS) and morphine simultaneously displayed a reduction in preference for morphine, while mice treated with ribozyme after inducing morphine dependence exhibited a preference for morphine.^47^, ^48^ This is likely mediated in part via an extracellular signal‐regulated kinase (ERK)‐dependent mechanism.^49^ These data suggest that morphine dependence may be prevented by interfering with Per1 gene expression. Additionally, morphine withdrawal led to desynchronization from the SCN circadian rhythm in rats.^50^ These studies offer new insights into molecular mechanisms underlying opioid tolerance, dependence, and withdrawal, and the identification of altered networks of genes involved in opioid‐related behaviors may provide potential targets to suppress opioid‐induced tolerance and dependence.

### 6.2 Effect of opioids on the circadian clock

#### 6.2.1 Effect on SCN neuronal excitability

Neurons in the SCN are thought to encode time of day by altering their excitability.^51^ Current evidence demonstrates circadian variations in the spontaneous excitability of SCN neurons, with increased neuronal excitability during the day and reduced neuronal excitability at night.^52^ In this regard, several ion channels including K^+^ channels, as well as voltage‐dependent and ‐independent Na^+^ and Ca^2+^ channels have been recognized to enhance neuronal excitability during the day, while certain K^+^ channels, including Ca^2+^‐activated BK channels, contribute to decreased neuronal excitability at night.^52^ These findings are supported by the study that showed spontaneous c‐fos expression, a neural activity marker, in the SCN early in the day.^53^

An effect of opioids on neuronal activity within the SCN has been shown in previous studies. For example, fentanyl (a potent μ‐opioid receptor agonist) was found to decrease electrical activity in the hamster SCN.^54^ Conversely, μ‐ or κ‐opioid receptor activation induced the neuronal expression of c‐fos in the SCN in rats.^53^ However, there is also evidence that suggests opioid receptor agonists are largely devoid of effect on SCN neuronal activity.^55^, ^56^, ^57^, ^58^ For example, some studies have reported that μ‐ and δ‐opioid receptors are expressed in low or undetectable levels within the mouse and rat SCN.^55^, ^56^, ^57^, ^58^ Furthermore, in an in vitro study, SCN neurons were mainly unresponsive to opioid receptor agonist morphine and enkephalins.^59^ These data suggest that opioids may partly affect the SCN circadian pacemaker activity indirectly via regulating the activity of projection neurons to the SCN.

Retinal ganglion cells, specifically intrinsically photosensitive retinal ganglion cells, project directly to the SCN via the retinohypothalamic tract and play an important role in the entrainment of circadian rhythms to light cues.^60^ DAMGO (a synthetic, highly selective μ‐opioid receptor agonist) was found to inhibit the firing activity of intrinsically photosensitive retinal ganglion cells and thereby suppress the transmission of environmental light information to the SCN.^60^ It is also probable that the inner geniculate leaflet (IGL) of the thalamus at least partly mediates opioid effects on circadian pacemaker activity.^43^, ^61^, ^62^, ^63^, ^64^ The IGL is a flat retinorecipient thalamic nucleus and plays a key role in regulating circadian rhythms.^64^ The IGL contains two main subpopulations of neurons: enkephalinergic neurons, which connect the leaflets located in both hemispheres, as well as neuropeptide Y (NPY)‐producing neurons that project to the SCN.^64^ In rats, enkephalins were found to suppress synaptic transmission in IGL neurons and strongly hyperpolarize these neurons via μ‐ and δ‐opioid receptors.^64^ The δ‐opioid receptors were also found presynaptically on axon terminals in the hamster SCN and IGL.^43^

**FIGURE 2:**
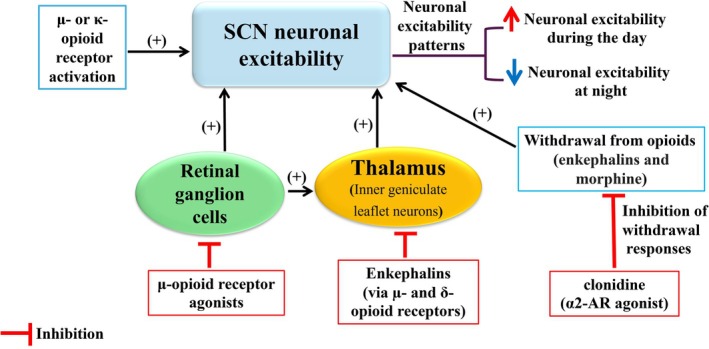
Effect of opioids on SCN neuronal excitability. Suprachiasmatic nucleus (SCN) neurons show a 24‐h rhythm in their firing rate, with higher rates during the day and lower rates at night. Opioid agonists have stimulatory effects on the firing rates of SCN neurons. Inputs from the thalamus and retinal ganglion cells also activate SCN neurons. Moreover, opioid withdrawal can lead to an excitatory response in some SCN neurons. This response is suppressed by clonidine (an α2‐AR agonist), suggesting a possible interaction between opioid receptors and α2‐adrenoceptors within the SCN.

Withdrawal from opioids (enkephalins and morphine) was also shown to activate SCN neurons in hamsters. These withdrawal responses were suppressed or attenuated by clonidine (an adrenoceptor agonist).^59^ The effect of opioids on SCN neuronal excitability has been shown in Figure 2.

#### 6.2.2 Effect of opioids on the circadian clock early in life

Early life opioid exposure appears to influence the maturation of the circadian system. In this regard, repeated exposure of rat dams to methadone (a synthetic opioid agonist used for the treatment of opioid use disorders during pregnancy) during lactation and pregnancy significantly decreased the rhythm of aralkylamine N‐acetyltransferase (NAT; a key enzyme in the biosynthesis of melatonin) activity in the pineal gland of offspring.^65^ In another study, maternal morphine intake during pregnancy and lactation increased Per2 (but not Per1) gene expression in the SCN and caused arrhythmicity in NAT activity in the pineal gland of rat pups.^66^

The interaction between opioids and the circadian clock is summarized in Table 3.

**TABLE 3:** Summary of the interaction between opioids and the circadian clock.

Category	Effect on the circadian/opioid systems
Per2 (Brdm1) mutant mice	A decrease in morphine tolerance and withdrawal signs compared with wild‐type control mice
Simultaneously treatment of mice with ribozyme (to interfere with mPer1 gene expression in the CNS) + morphine	A reduction in preference for morphine
Mice treated with ribozyme after inducing morphine dependence	Preference to morphine
Morphine withdrawal in rats	A decrease in the expression of Per1 and Per2 genes in mesolimbic brain regions in rats
Repeated exposure of rat dams to methadone during lactation and pregnancy	Decrease in the rhythm of aralkylamine N‐acetyltransferase activity in the pineal gland of offspring
Maternal morphine intake during pregnancy and lactation	An increase in Per2 (but not Per1) gene expression in the SCN of rat pups. Arrhythmicity in aralkylamine N‐acetyltransferase activity in the pineal gland of rat pups.

## 7 INTERACTION BETWEEN SLEEP/WAKE, CIRCADIAN RHYTHMS, AND THE OPIOID SYSTEM

Opioid receptors are found in multiple brain regions involved in sleep regulation, including the hypothalamus, the dorsal raphe nucleus, and the LC, and thus have the potential to contribute to opioid‐induced alterations in sleep/wake behaviors.^2^ This is supported by the studies that indicate chronic opioid use causes sleep problems,^67^, ^68^, ^69^, ^70^ which have been shown to be a notable risk factor for drug abuse, relapse, and mental disorders.^71^, ^72^ Opioid‐induced sleep problems were also found to aggravate existing pain symptoms in patients with chronic pain,^73^ and inadequate pain control and treatment may result in relapse.^74^ Given the importance of sleep in the successful treatment of both opioid use disorders and pain, understanding the mechanisms underlying opioid‐induced disruption of healthy sleep/wake behaviors seems to be essential.^58^

**FIGURE 3:**
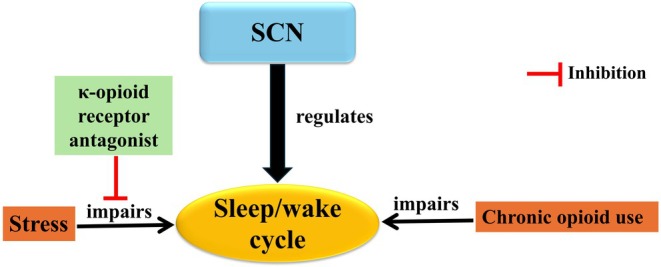
Interaction between sleep/wake, circadian rhythms, and the opioid system. The suprachiasmatic nucleus (SCN) regulates the timing of the sleep–wake cycle. Chronic opioid use is linked to sleep disturbances. κ‐opioid antagonists could improve stress‐induced sleep/wake cycle disturbance.

The occurrence of sleep and waking has been shown to be regulated by homeostatic and circadian processes.^75^ Homeostatic sleep is regulated by the longer that an organism stays awake, while circadian sleep is often affected by external factors, particularly light.^76^ Previous studies revealed that light, via intrinsically photosensitive retinal ganglion cells, regulates sleep and wake states independent of image formation.^76^, ^77^, ^78^ It has been shown that intrinsically photosensitive retinal ganglion cells project to the SCN of the hypothalamus to synchronize sleep and wake cycles to environmental light.^76^, ^78^, ^79^, ^80^ There is evidence that suggests opioids could alter the activity of intrinsically photosensitive retinal ganglion cells in both humans and rodents. Intrinsically photosensitive retinal ganglion cells (M1–M3 subtypes) were found to express μ‐opioid receptors in rodents.^60^ Selective agonists of μ‐opioid receptors and selective antagonists or genetic ablation of these receptors from retinal ganglion cells were demonstrated to eliminate and enhance, respectively, the pupillary light reflex mediated by intrinsically photosensitive retinal ganglion cells.^81^ Also, M1 intrinsically photosensitive retinal ganglion cells project to several brain areas involved in the circadian entrainment of sleep and wake states to light including the SCN, as well as the ventral lateral geniculate nucleus and the intergeniculate leaflet of the thalamus.^61^, ^62^, ^63^ Thus, intrinsically photosensitive retinal ganglion cells have been suggested to be a potential therapeutic target for the treatment of opioid‐induced sleep disorders.^58^ In addition to the μ‐opioid receptors, the κ‐opioid receptors could influence the sleep/wake rhythms. For example, the administration of a κ‐opioid receptor antagonist before stress induction improved stress impacts on disrupted sleep and circadian rhythms and diminished alterations in mPer2 gene expression in the nucleus accumbens (NAc),^82^ a brain region involved in the sleep/wake regulation. The interaction between sleep/wake, circadian rhythms, and the opioid system has been shown in Figure 3.

## 8 INTERACTION BETWEEN MELATONIN, CIRCADIAN RHYTHMS, AND OPIOIDS

Melatonin is a hormone produced and released chiefly by the pineal gland and has been shown to control the circadian timing of the SCN.^83^ The pineal gland is connected to the SCN through a multi‐synaptic pathway: circadian signals are transmitted from the SCN to the PVN, then to the intermediolateral nucleus of the spinal cord, to the sympathetic superior cervical ganglion, and finally to the pineal gland.^84^ Light, via the retinohypothalamic tract, directly stimulates the SCN. In addition, retinal ganglion cells seem to indirectly project to the pineal gland and inhibit melatonin synthesis.^85^ Early life experiences have been demonstrated to affect the developing circadian system and thus the diurnal rhythm of melatonin synthesis. For example, complete lesions of the SCN in pregnant rats perturbed rhythms of SCN glucose utilization in fetuses and pineal NAT (an enzyme involved in the diurnal rhythm of melatonin synthesis) activity in 10‐day‐old offspring.^86^

In both humans and animals, melatonin is produced in a circadian manner during darkness while light inhibits melatonin generation and release.^87^, ^88^ A nocturnal peak in plasma and cerebrospinal fluid (CSF) melatonin levels has been shown in humans.^89^ Similarly, Wistar rats exhibited a nocturnal peak in plasma and pineal gland melatonin levels.^86^ Circadian melatonin secretion in serum and urine is regarded as a biomarker for disrupted circadian rhythm and has been associated with sleep disorders.^90^ The activity of NAT, which is crucial for melatonin synthesis, is also highly rhythmic, peaking at night and reducing during the day.^91^ This rhythm is affected by light–dark cycles, with light inhibiting NAT activity.^91^ This rhythmic activity is an essential indicator of the timing of melatonin synthesis, which is vital for regulating sleepiness and sleep onset.^92^ Hence, investigating the circadian rhythm of NAT activity could be important for understanding how melatonin regulates sleep–wake cycles.

There are three melatonin receptors, MT1, MT2, and MT3, which are a type of GPCRs. Activation of the MT1 melatonin receptor was found to suppress neuronal activity in the SCN, and the MT2 melatonin receptor can shift circadian rhythms produced in the SCN.^93^

Melatonin has been shown to contribute to the regulation of neuropathic pain, indicating significant analgesic effects.^94^ Melatonin can also act as a potential enhancer of the antinociceptive effect of certain opioids.^95^, ^96^, ^97^ For example, the co‐treatment of melatonin with a low dose of morphine resulted in a brief antinociceptive impact.^96^ Melatonin also augmented the antinociceptive response of morphine in the formalin model of pain in mice.^97^ Additionally, the co‐administration of melatonin with morphine reversed morphine‐induced tolerance and dependence in mice.^98^

Melatonin partly mediates effects of natural light cycles on opioid‐mediated behaviors. For instance, a decrease in melatonin levels in rats exposed to continuous brightness was associated with increased consumption of morphine and more severe morphine withdrawal symptoms.^99^ In another study, disrupted circadian light exposure markedly enhanced morphine tolerance and attenuated the antinociceptive effect of morphine in a rat model of neuropathic pain, while melatonin co‐treatment reduced morphine tolerance in the animals.^100^ These data propose that a proper circadian light control in patients receiving chronic opioid therapy may help patients to entrain normal circadian rhythms and prevent, in part, opioid tolerance and dependence. Accordingly, melatonin may exert its analgesic effects, at least in part, through regulating the circadian rhythm of the endogenous opioid system as well as the synthesis of the endogenous opioids in the brain. For example, pinealectomy abrogated the 24‐h rhythm of morphine analgesia^101^ and disturbed the diurnal rhythm in Met‐enkephalin content in the rat hypothalamus.^102^ In another study, pinealectomy disrupted the opioid peptide circadian rhythm accompanied by a reduction in the tissue content of enkephalin peptides (Met‐enkephalin, Leu‐enkephalin and Synenkephalin) in the hypothalamus and hippocampus of rats, while these events were reversed following treatment with exogenous melatonin and darkness presentation.^38^

**FIGURE 4:**
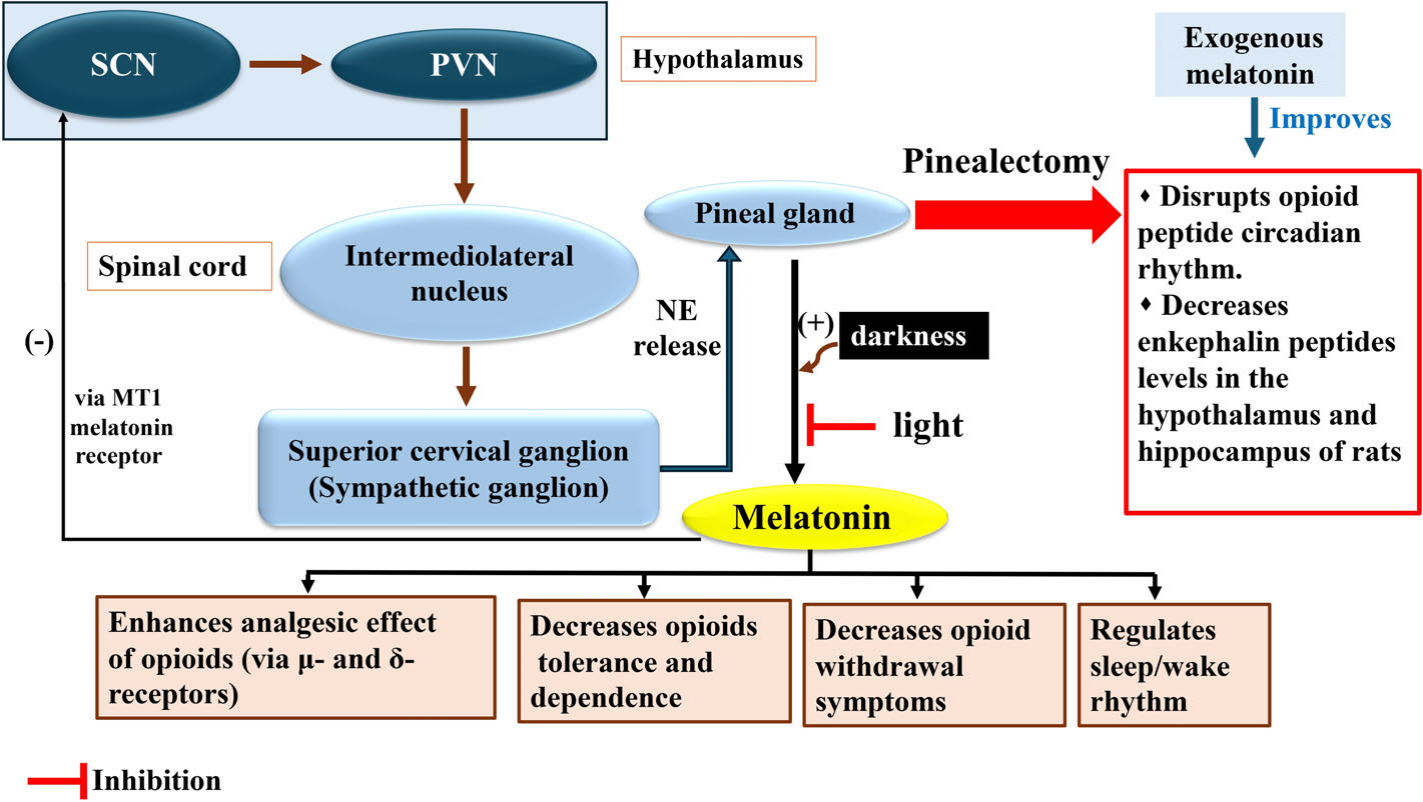
Interaction between melatonin, circadian rhythms, and opioids. Melatonin production is controlled by a circadian pathway involving the SCN, paraventricular nucleus (PVN), and intermediolateral nucleus. The SCN transmits signals to the PVN, which then projects to the intermediolateral nucleus in the spinal cord. These projections ultimately affect sympathetic outflow to the pineal gland (NE (norepinephrine) release), where melatonin is produced and released. Light inhibits melatonin synthesis, while darkness promotes it. Melatonin can interact with opioids in ways that can be beneficial. It can increase opioid analgesia and reduce tolerance and dependence as well as withdrawal symptoms associated with opioid use.

Although the exact mechanisms by which circadian hormone melatonin attenuates certain adverse effects of chronic opioid use are still unknown, a reduction in pro‐inflammatory and pain‐associated receptor genes, upregulation of the Kcnip3 gene (potassium voltage‐gated channel interacting protein 3; Kcnip3 which plays a role in the regulation of pain sensitivity and anxiety‐ and depression‐like behaviors^103^), and enhanced levels of antioxidative enzymes have been proposed.^100^ Melatonin might also influence opioid‐mediated behaviors through regulating the sleep/wake rhythm,^83^ as the circadian sleep/wake cycle is tightly regulated by melatonin and its disruption has been reported to promote opioid craving and relapse.^104^, ^105^ While some studies suggest a role of μ‐opioid receptors in melatonin‐dependent analgesic impacts,^106^ other studies demonstrate that the δ‐opioid receptors, rather than the μ‐ opioid receptor, mediate the antinociceptive impacts of melatonin in the brain.^107^ The interaction between melatonin, circadian rhythms, and opioids has been shown in Figure 4.

## 9 INTERACTION BETWEEN DOPAMINERGIC NEUROTRANSMISSION, CIRCADIAN RHYTHMS, AND OPIOIDS

Dopamine (DA) is a key neurotransmitter in the brain's reward system. The experience of reward is associated with activation of the mesocorticolimbic DA circuitry.^108^ The mesolimbic and mesocortical pathways are two major DAergic pathways in the brain that principally originate in the ventral tegmental area (VTA). The mesolimbic pathway projects to the hippocampus, amygdala, and NAc (a main component of the ventral striatum), while the mesocortical pathway projects to cortical areas including the prefrontal cortex, anterior cingulate cortex, and orbitofrontal cortex. Previous studies have revealed an essential role of the mesocorticolimbic DAergic system in mediating the motivational impacts of opioids. In this respect, opioids interact with the mesolimbic pathways via binding with their receptors and thereby affect mesolimbic DA, principally in the NAc.^109^ In one study, opioids such as morphine or heroin (μ‐ receptor agonists) were found to bind with GPCRs and activate DA release by dopamine‐containing neurons in the VTA indirectly through GABAergic disinhibition.^110^ In another study, stimulation of different opioid receptor types produced opposing effects on mesolimbic DA. For example, the stimulation of μ‐opioid receptors in the VTA was indicated to enhance DA release, while κ‐opioid receptor stimulation in the NAc was found to decline DA release.^111^

In addition to reward stimuli, aversive stimuli, such as pain, also elicit DA release in the brain^112^, ^113^ and there is evidence that suggests an essential role of mesolimbic DA in the suppression of tonic pain. For example, DA depletion using 6‐hydroxydopamine lesions within the ventral midbrain suppressed morphine analgesia in the formalin inflammatory test in animals.^114^ A reduction in DA release in the NAc following chronic stress exposure was also associated with the development of long‐term hyperalgesia.^115^ Moreover, stimulation of the D2 receptor, but not the D1 receptor, in the NAc suppressed the persistent phase of formalin‐induced nociception.^116^ A reduction in D2 receptor binding^117^, ^118^, ^119^, ^120^ and presynaptic DA activity^121^, ^122^ has also been shown in patients with chronic pain. These data suggest a possible role of DA D2 receptor agonists in pain suppression. This is further supported by a growing body of evidence showing that DA, by acting on D2 receptors, exerts an antinociceptive effect.^123^, ^124^, ^125^, ^126^, ^127^

It has been demonstrated that opioid withdrawal results in a disruption of DAergic signaling.^128^ Chronic opioid exposure was also found to induce a hypodopaminergic state, leading to compulsive drug seeking.^129^, ^130^, ^131^ Several lines of evidence also demonstrated that chronic pain results in a hypodopaminergic state.^132^ Thus, considering a role of DA in attenuating chronic pain and that a hypodopaminergic state is thought to induce hyperalgesia,^115^ restoring DA signaling may help pharmaceutical opioid analgesic agents alleviate chronic opioid‐induced hyperalgesia and withdrawal.

Among the clock genes are those that have been indicated to affect the function of the mesolimbic dopaminergic system involved in reward processing and the modulation of addictive‐related behaviors.^133^, ^134^, ^135^, ^136^, ^137^ Chronic morphine withdrawal was found to reduce the expression levels of Per2 and Per3 genes in the NAc of rats.^138^ Moreover, circadian gene disruption in the NAc was associated with altered pain sensation in rodents. For instance, long‐term nitroglycerin therapy resulted in hind paw and cephalic hyperalgesia in mice accompanied by overexpression of Per3 in the NAc and trigeminal ganglion,^139^ both of which are involved in pain circuitry.^140^

**FIGURE 5:**
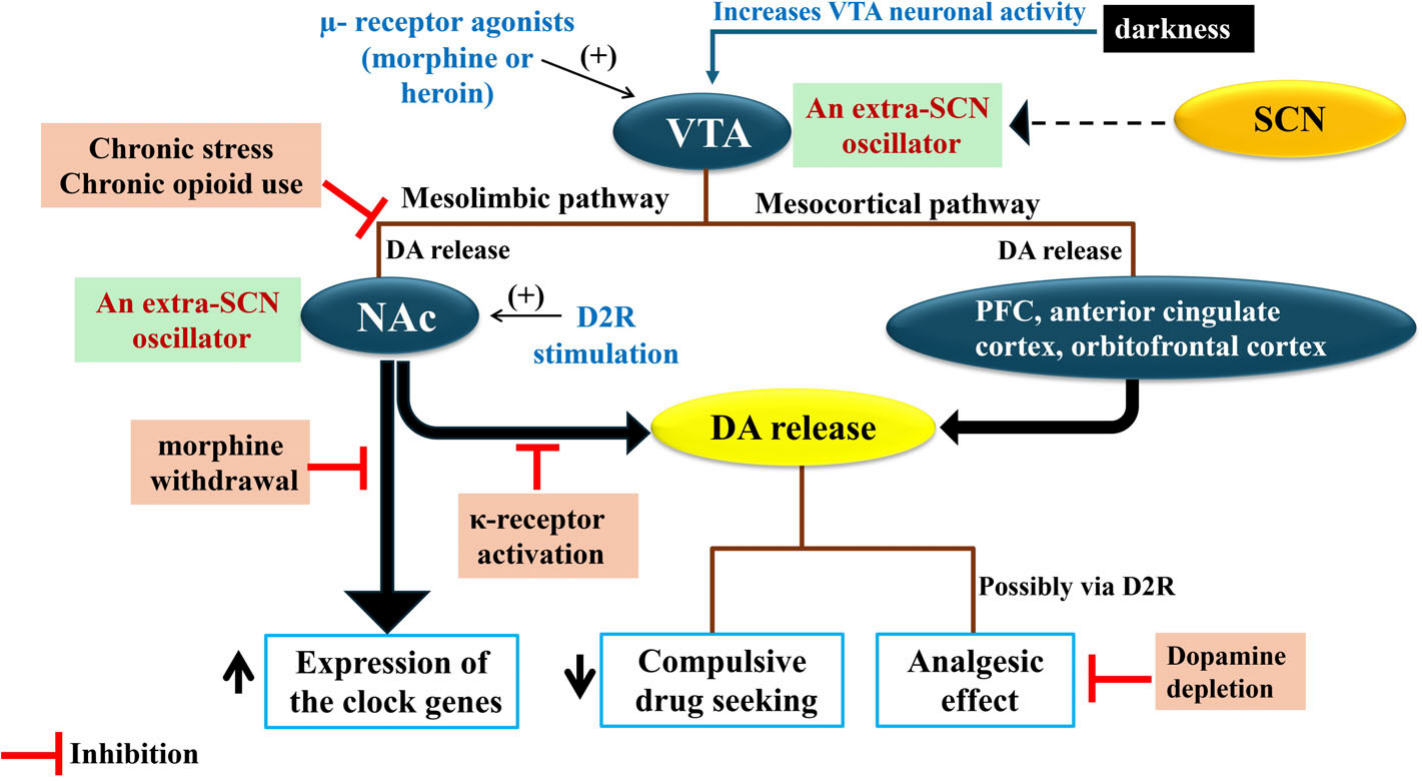
Interaction between dopaminergic neurotransmission, circadian rhythms, and opioids. Opioids, particularly via activation of μ‐opioid receptors, indirectly affect the mesolimbic dopamine system, resulting in enhanced dopamine (DA) release and reinforcing impacts. Conversely, dopamine receptors, especially D2 receptors, can modulate the opioid system. The ventral tegmental area (VTA) and the nucleus accumbens (NAc) are considered extra‐SCN circadian oscillators. They exhibit circadian rhythms in function, activity, and molecular clock function. The NAc and VTA may receive and integrate circadian information via indirect connections with the SCN. The dotted arrow indicates indirect effects.

In addition to the SCN circadian pacemaker, the VTA and NAc are considered extra‐SCN oscillators, and their inherent circadian properties seem to affect drug abuse, reward processing, and susceptibility to develop substance use disorders. Single‐unit extracellular recordings in rats revealed a rhythmic pattern of VTA DA neuronal activity and a higher D2 autoreceptor responsiveness in the dark phase.^141^ Multi‐unit activity recordings in mice also showed a potent circadian rhythm of VTA activity, with higher VTA neuronal activity during the animal's active phase (dark phase).^142^ In addition to the VTA neuronal activity, DA signaling, DA levels, and its metabolites were found to diurnally vary in the NAc.^143^, ^144^, ^145^, ^146^, ^147^, ^148^ Previous studies also revealed circadian variations in the expression of DA receptors (e.g., D_1_, D_2_, and D_3_ receptors) in the NAc.^3^, ^148^ Altered molecular rhythms were also shown in the NAc of people with opioid use disorder, with nearly double the number of rhythmic transcripts in subjects with opioid use disorder compared to unaffected subjects.^149^ The rhythmic activity of the VTA and NAc seems to be due to both local circadian molecular clock function in these areas and entrainment by indirect inputs from the SCN.^3^ Together, these data suggest that a proper circadian molecular clock function in the VTA and NAc may influence chronic opioid‐induced adverse effects. The interaction between dopaminergic neurotransmission, circadian rhythms, and opioids has been shown in Figure 5.

## 10 INTERACTION BETWEEN SEROTONERGIC NEUROTRANSMISSION, CIRCADIAN RHYTHMS, AND OPIOIDS

Serotonergic neurons in the CNS (e.g., dorsal raphe and median raphe nuclei) are distributed in the brainstem and provide innervation to the brain.^150^ 5‐HT plays a critical role in regulating sleep, mood, and emotions. There are several 5‐HT receptor subtypes including 5HT1 (5HT1A, 5HT1B, 5HT1D, 5HT1E, and 5HT1F), 5HT2 (5HT2A, 5HT2B, and 5HT2C), 5HT3, 5HT4, 5HT5, 5HT6, and 5HT7 receptors. Except for the 5HT3 receptor, which is a ligand‐gated ion channel, other 5‐HT receptors are GPCRs.^150^, ^151^

Previous research showed a role of serotonin in the modulation of morphine dependence.^152^ It is also reported that negative affect associated with protracted opiate withdrawal is mediated by serotonin signaling.^153^ For instance, morphine withdrawal was associated with enhanced 5‐HT turnover, particularly in the dorsal raphe, and the selective serotonin reuptake inhibitor fluoxetine reversed morphine withdrawal‐induced immobility and sociability deficits in the tail suspension test (a measure of depressive‐like behavior in rodents).^153^ Moreover, it has been demonstrated that morphine withdrawal‐induced sociability deficits need tumor necrosis factor‐α (TNF‐α) release from the lateral habenula projecting to the dorsal raphe.^154^

Although studies examining direct interactions between serotonergic neurotransmission, chronic opioid‐mediated behaviors, and circadian rhythms have not been conducted, a number of indirect studies suggest the existence of a relationship. Both the dorsal raphe nucleus and median raphe nucleus project to the SCN and elicit 5‐HT release in the SCN.^155^, ^156^ Electrical stimulation of both nuclei has been shown to decrease light‐induced activity in the SCN and cause circadian rhythm phase shifts,^156^ likely via 5‐HT7 receptor.^157^ Moreover, non‐selective 5‐HT antagonists and 5‐HT1A antagonists were demonstrated to augment light‐induced increases in SCN neuronal firing rate.^158^ Both serotonin and 5‐HT1A agonists were also found to suppress optic nerve‐induced field potentials recorded in the SCN in the hypothalamic slice preparation. 5‐HT1A agonist also blocked light‐induced fos expression in the SCN.^159^ These data suggest a possible role of serotonin in tonic inhibition of light inputs to the SCN by acting on 5‐HT1A. Another study demonstrated that 5‐HT2C receptor activation can mimic the impacts of light on SCN neurons and melatonin synthesis in rats.^160^ These data might result in the development of new pharmacological tools to correct rhythm disruptions in humans.

**FIGURE 6:**
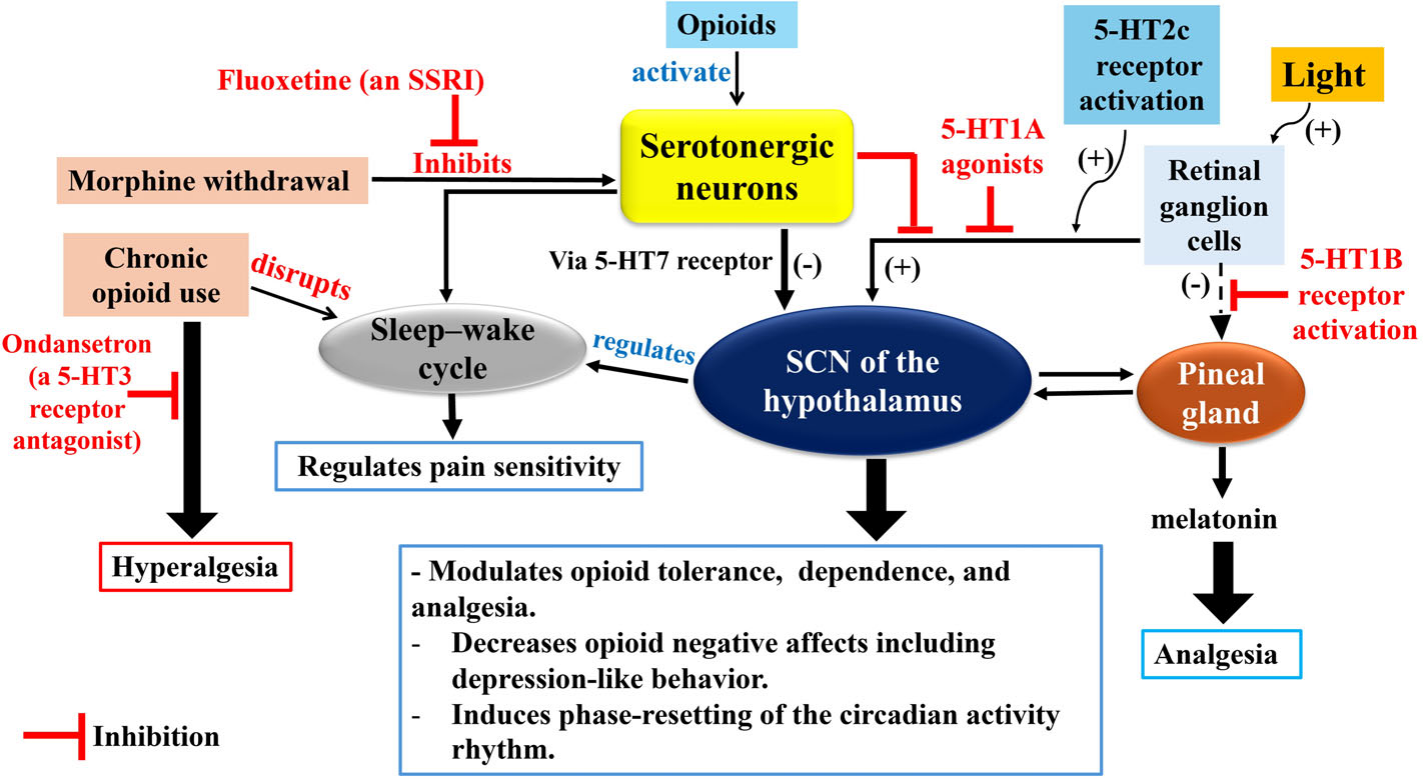
Interaction between serotonergic neurotransmission, circadian rhythms, and opioids. Serotonin modulates opioid tolerance, dependence, and analgesia as well as opioid withdrawal. It regulates sleep–wake cycles, affects the sensitivity of the circadian clock to light, and has a significant role in resetting the circadian activity rhythm. Also, serotonin, by acting on the 5‐HT7 receptor, can reduce suprachiasmatic nucleus (SCN) neuronal activity. Different types of serotonin receptors have varying impacts on opioid effects; serotonin receptors 5‐HT3 can reduce opioid analgesia, while 5‐HT1B can enhance opioid analgesia. The dotted arrow indicates indirect effects.

Serotonergic neurons in the dorsal raphe nucleus play a significant role in the regulation of the sleep–wake cycle,^161^, ^162^ and their disruptions have been shown to aggravate pain symptoms in chronic pain patients.^73^ Also, chronic morphine‐induced hyperalgesia is accompanied by disrupted circadian rhythm,^140^ and the 5‐HT3 receptor is presumed to play a significant role in morphine‐induced hyperalgesia.^163^ Administration of ondansetron, a 5‐HT3 receptor antagonist, also prevented the development of chronic morphine‐induced hyperalgesia.^163^ Additionally, 5‐HT1B receptor activation was associated with a reduction in the inhibitory impact of light on pineal melatonin generation.^164^ As mentioned above, melatonin regulates both the synthesis and the circadian rhythm of the endogenous opioids,^101^, ^102^ increases the analgesic effect of opioids,^97^ and reduces opioid withdrawal and tolerance.^99^, ^100^ Considering that serotonergic neurons in the raphe nucleus express opioid receptors and directly respond to opioids,^2^, ^165^ the mentioned studies point towards the idea that serotonin may be involved in the modulation of opioid tolerance, dependence, and analgesia by circadian rhythms. The interaction between serotonergic neurotransmission, circadian rhythms, and opioids has been shown in Figure 6.

## 11 INTERACTION BETWEEN NORADRENERGIC NEUROTRANSMISSION, CIRCADIAN RHYTHMS, AND OPIOIDS

In the brain, norepinephrine (NE) originates from two noradrenergic nuclei in the brainstem: the LC and the solitary tract (NTS). Among these nuclei, LC is the principal source of NE and projects extensively throughout the brain. The NTS receives visceral sensory information and modulates autonomic responses to stress.^166^ NE is involved in a variety of behavioral and physiological processes including arousal, attention, learning and memory, and stress response.^167^ It serves its effects via binding to G‐protein‐coupled α‐ and β‐adrenergic receptors (ARs), which are further divided into α1‐AR (α1A, α1B, and α1D), α2‐AR (α2A, α2B, and α2C), and β‐ARs (β1, β2, and β3) subtypes. NE has a higher affinity for α2‐ARs than β‐ARs.^167^

Previous studies have reported circadian rhythms in NE storage and synthesis, as well as in tyrosine hydroxylase activity (the rate‐limiting enzyme of NE synthesis) in the brain, with a peak during the dark period.^168^ NE has been shown to affect circadian rhythms in mammals. In this regard, NE, by acting on β2‐AR, elicited transient expression of Per1 in astrocytes.^169^ In another study, NE administration induced Per1 mRNA expression in the cerebral cortex of mice.^170^ Furthermore, Per3 expression was higher in control samples incubated with NE than controls without NE, while Per3 expression was lower in cultures from ADHD patients with no NE than controls with NE.^171^ ADHD is linked to cognitive deficits, and NE has been shown to play a crucial role in these deficits.^172^ These data suggest that opioid‐related cognitive deficits may partly involve disrupted circadian rhythms in NE.

The pineal gland receives adrenergic innervation, which is vital for natural nighttime generation of melatonin.^173^ Studies have shown a diurnal variation in the expression of β‐ARs in the pineal gland. These rhythmic variations are regulated by the SCN, which stimulates NE release from pineal sympathetic afferents (from the superior cervical ganglion) during the night. NE binds to β‐ARs and increases pineal melatonin production. In the rat pineal gland, the density of β‐ARs reaches its peak either late in the light phase or at mid‐dark.^174^ Additionally, NE synchronization of rat pineal gland culture was indicated to maintain the natural rhythmic release of NE, enhancing melatonin production.^175^ This NE release in the pineal gland can be strongly suppressed by light.^84^

The α1‐, α2‐, and β‐ARs have been identified on thalamic IGL neurons,^63^ which in turn send integrated information synchronizing circadian rhythms to the SCN as well as express both μ‐ and δ‐opioid receptors.^64^ Furthermore, previous studies have shown that μ‐ and δ‐opioid receptor agonists increase NAT activity and thereby boost the synthesis of melatonin,^176^ which plays a crucial role in regulating circadian rhythms and influences opioid effects. These data suggest the existence of an interplay between the adrenergic, circadian, and opioid systems. This idea is supported by an in vitro study in which activation of SCN neurons by enkephalins and morphine was inhibited or attenuated by the α2‐AR agonist clonidine.^59^

The NE system plays a significant role in the stress response via an effect on the hypothalamus–pituitary–adrenal (HPA) axis, which in turn interacts with both the circadian clock and the opioid system.

## 12 INTERACTION BETWEEN STRESS RESPONSE, CIRCADIAN RHYTHMS, AND OPIOIDS

The HPA axis is a critical component of the stress response in the CNS and acts synergistically with the LC to cope effectively with stressful conditions. In response to stressors, neurons located in the paraventricular nuclei (PVN) of the hypothalamus are activated and release corticotropin‐releasing hormone (CRH) and arginine vasopressin (AVP) into the anterior lobe of the pituitary, which act synergistically to trigger the synthesis and release of adrenocorticotropic hormone (ACTH) into circulation. In turn, the increased circulating ACTH concentrations cause glucocorticoid steroid hormones to be released from adrenal glands.^177^

The stress system is known to be closely related to the circadian clock, and the reciprocal interactions between the stress and circadian systems are shown to play an essential role in the preservation of physiological homeostasis. Disruptions in both the circadian and stress systems appear to be involved in substance use disorder and addiction.^178^, ^179^, ^180^

### 12.1 Stress effect on circadian rhythm

Stress has been shown to affect the rhythmic expression of circadian genes. In this respect, acute and chronic stress were found to enhance levels of circadian gene Per1 in some neural and peripheral tissues.^181^, ^182^, ^183^ Per2 expression was also induced in response to stress^184^, ^185^ and even lost its rhythmic expression in the bed nucleus of the stria terminalis and in the central nucleus of the amygdala following adrenalectomy.^186^ It was also demonstrated that glucocorticoid receptors are needed for the rhythmic expression of Per2.^187^ Both Per1 and Per2 genes contain a glucocorticoid‐responsive element in their promoter areas,^185^, ^188^ which may mediate glucocorticoid effect on alterations in their rhythmic expression.^189^ While the effects of acute stress on changes in gene expression may be transient, chronic stress and the long‐term presence of glucocorticoids have deleterious consequences and even may lead to clock entrainment.^189^, ^190^

### 12.2 Opioid effect on stress response

The κ‐opioid receptor activation and its endogenous opioid peptide, dynorphin, have been implicated in stress regulation.^191^, ^192^ For example, repeated social defeat stress for 3 days resulted in immobility and analgesia, while mice injected with the κ‐opioid receptor antagonist norBNI and prodynorphin gene disruption mice did not exhibit these behaviors.^193^ Moreover, the κ‐opioid receptor antagonist JDTic diminished chronic social defeat stress‐induced sleep and circadian rhythm disturbances as well as stress‐induced changes in mPer2.^82^

Previous experiments have shown a stimulatory effect of μ‐ and κ‐opioid receptor agonists on the HPA axis and the release of ACTH and corticosterone,^194^, ^195^, ^196^, ^197^, ^198^, ^199^ although some studies have reported an inhibitory effect of opioids on CRH, AVP, and noradrenergic neurons.^200^, ^201^ An opioid‐induced activation of the HPA axis is mediated, in part, directly via an effect on the secretion of hypothalamic CRH,^53^, ^202^ or indirectly via stimulating the activity of noradrenergic neurons innervating the PVN, the site of CRH‐secreting neurons.^203^, ^204^

### 12.3 Role of the NE system

A role of noradrenergic neurons in stimulating the HPA is supported by the observation that noradrenergic neurons from the NTS predominantly project to CRF‐secreting neurons of the PVN^205^, ^206^ and that the stimulatory effect of morphine on the HPA axis activity was suppressed by α‐ and β‐adrenoceptor antagonists.^203^ Moreover, μ‐opioid receptors have been demonstrated to play a pivotal role in modulating the central stress response via suppressing NE release from the LC, leading to the inhibition of CRH release from the PVN.^10^ LC neuronal activity, and thus NE release, is principally regulated by CRH and endogenous opioids, with CRH exerting an excitatory impact and opioids exerting an inhibitory impact.^207^

Together, endogenous opioids (principally enkephalin and endorphin) are released in response to stress. Although some conflicting results show a stimulatory effect of opioids on the HPA axis, there is potent evidence showing that endogenous opioids attenuate or terminate stress responses partly by opposing actions induced by CRH.^207^

### 12.4 Circadian regulation of the HPA axis and role of opioids

**FIGURE 7:**
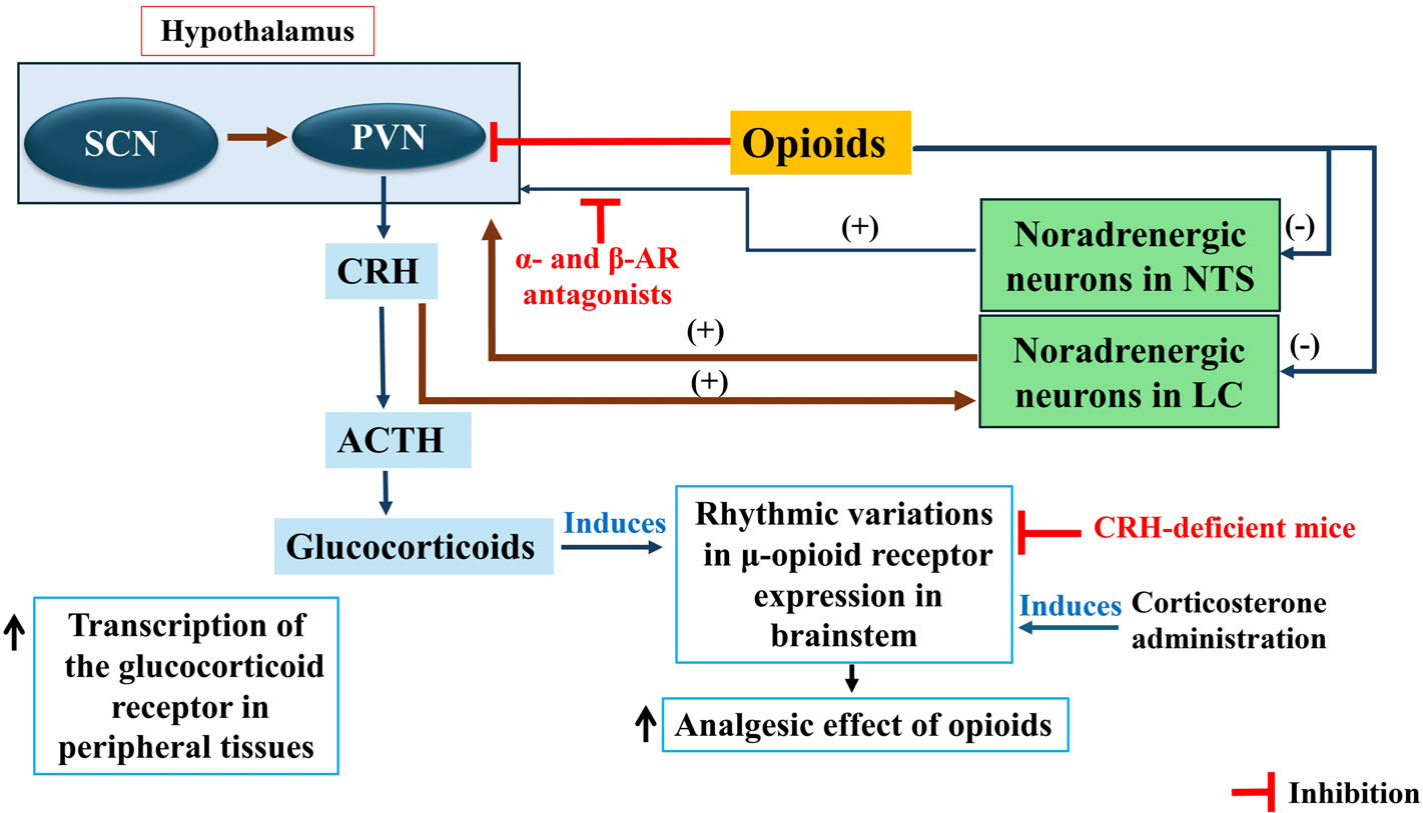
Interaction between stress, circadian rhythms, and opioids. The hypothalamic–pituitary–adrenal (HPA) axis, opioid system, and adrenergic system are activated during a stress response. The stress response involves a complex interplay between these systems. The suprachiasmatic nucleus (SCN) exerts an indirect influence on the release of CRH, adrenocorticotropic hormone (ACTH), and glucocorticoids, primarily through the hypothalamic–pituitary–adrenal (HPA) axis. The SCN sends signals to the paraventricular nucleus (PVN) of the hypothalamus, which triggers the release of CRH. CRH then stimulates the anterior pituitary to release ACTH, which in turn causes the adrenal cortex to secrete glucocorticoids. The SCN regulates the daily release of glucocorticoids, which in turn can influence the analgesic effects of opioids. The SCN indirectly (the dotted arrow indicates indirect effects) influences the transcription of the glucocorticoid receptor in peripheral tissues by regulating the fluctuating glucocorticoid level. Noradrenergic neurons in the nucleus of the solitary tract (NTS) and locus coeruleus (LC) play a crucial role in the stress response and interact with the HPA axis and opioids. Opioids inhibit the HPA axis activity, while adrenergic stimulation can activate it.

The HPA axis and its hormones (CRH, ACTH, and glucocorticoids)^189^ as well as noradrenergic neurons in the LC^40^, ^208^ are under circadian regulation by the SCN. Furthermore, the clock system has been shown to regulate the transcriptional activity of the glucocorticoid receptor in peripheral tissues.^209^ The rhythmic secretion of glucocorticoids was found to induce rhythmic variations in the expression of μ‐opioid receptor in mouse brainstem, which in turn could affect circadian variations in the pain threshold.^210^ In this regard, CRH‐deficient mice, which had a disruption in the circadian rhythm of glucocorticoid release, showed no variation in the expression of μ‐opioid receptor, while corticosterone administration induced μ‐opioid receptor expression in the brainstem and increased the analgesic effect of morphine in the animals.^210^ These data suggest that a potent analgesic impact of morphine may be achieved when μ‐opioid receptor function is enhanced. Considering a stimulatory effect of glucocorticoids on μ‐opioid receptor expression and that the 24‐hour rhythm of circulating glucocorticoids plays an important role in synchronizing central and peripheral clocks,^211^ therefore, chronopharmacological strategies that act to normalize the rhythm of circulating glucocorticoid levels may minimize complications and adverse impacts of chronic opioid treatment. The interaction between stress, circadian rhythms, and opioids has been shown in Figure 7.

## 13 INTERACTION BETWEEN OXIDATIVE STRESS, CIRCADIAN RHYTHMS, AND OPIOIDS

Oxidative stress refers to excess generation of reactive oxygen species (ROS) compared to antioxidant defense, resulting in oxidative damage and finally cell death, and has been linked to numerous pathologies. A role of oxidative stress in nociception has been shown in previous studies.^212^, ^213^, ^214^, ^215^, ^216^, ^217^ For example, ROS is involved in various types of hyperalgesia including mechanical^213^ and inflammatory hyperalgesia,^214^ N‐methyl‐D‐aspartate (NMDA)‐mediated hyperalgesia,^215^ as well as hyperalgesia induced by capsaicin^216^ and chemokine CCL2.^217^ Furthermore, in previous research, a superoxide dismutase (a strong antioxidant) mimetic suppressed all measured parameters of inflammation and hyperalgesia in carrageenan‐induced inflammatory hyperalgesia in rats.^218^

Evidence suggests an essential role of ROS in the development of opioid‐induced hyperalgesia and tolerance.^212^, ^219^, ^220^ Opioids have been shown to induce ROS production and impair the function of antioxidant enzymes including glutathione, glutathione peroxidase, superoxide dismutase, and catalase.^212^, ^221^, ^222^, ^223^ While the protective effect of melatonin on morphine‐induced tolerance in rats was associated with enhanced levels of these antioxidative enzymes in the spinal cord.^100^ Oxidative stress can impair the function of μ‐opioid receptors and proteins.^224^, ^225^ Moreover, an increase in age‐related oxidative stress was associated with a marked reduction in the nociceptive threshold and the antinociceptive impacts of opioids (morphine and fentanyl) in rats.^226^ Also, a substantial negative correlation between the antinociceptive impact of morphine and oxidative stress was shown in various brain areas of aging rats.^226^

Growing evidence suggests the existence of circadian rhythm connections to oxidative stress. For example, many antioxidants and enzymes that protect the cell against oxidative stress show circadian oscillations in their activity or expression.^227^ Moreover, protein oxidation, lipid peroxidation, protein damage, and DNA damage exhibit circadian rhythmicity.^227^, ^228^, ^229^, ^230^ These circadian variations in redox state are regulated by the clock genes, which have been shown to control basic cellular processes including cellular responses to DNA damage (i.e., repair and apoptosis).^231^, ^232^

**FIGURE 8:**
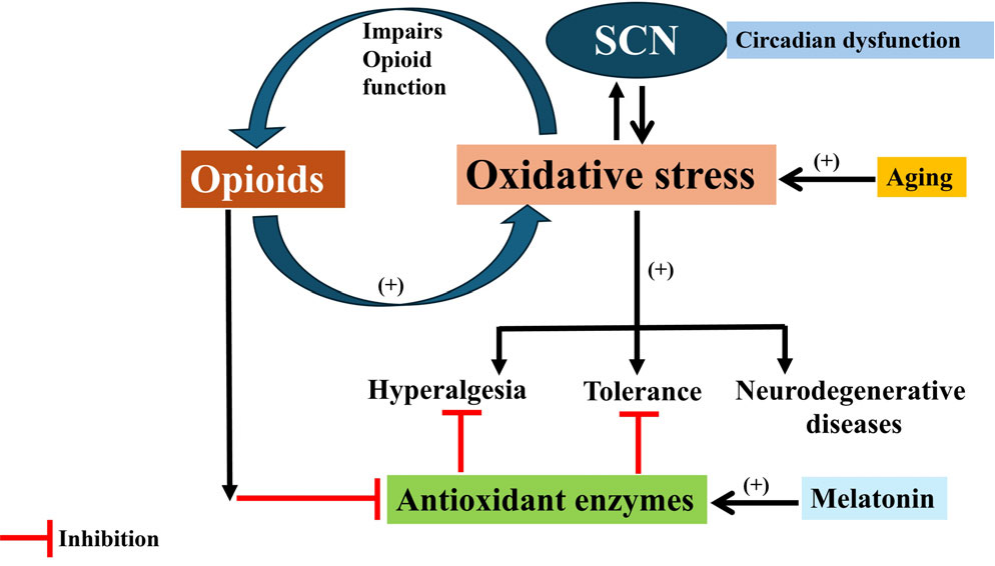
Interaction between oxidative stress, circadian rhythms, and opioids. Opioids can increase reactive oxygen species (ROS) and decrease antioxidant defenses, contributing to neurotoxicity, tolerance, and dependence. Oxidative stress can also impair the function of opioid receptors. This can influence the ability of opioids to bind and transmit signals, thus decreasing their analgesic impacts.

Circadian dysfunction has been proposed to contribute to neurodegenerative diseases partly via promoting oxidative stress.^233^ Circadian oscillations in redox state (the balance between oxidants and antioxidants) have also been shown to affect neuronal excitability in SCN neurons and circadian output in rats.^234^ Oxidative stress also resulted in circadian disruption,^235^ and disruption of the circadian system in mice lacking BMAL1 (a core clock gene) led to enhanced oxidative stress in several organs including the brain.^236^, ^237^ Circadian rhythms and oxidative stress are further connected by the role that they play in opioid‐induced hyperalgesia and tolerance. Considering these connections, a combinatorial approach consisting of antioxidants and approaches for resynchronization and normalization of circadian rhythms may be beneficial in attenuating opioid‐induced adverse effects. The interaction between oxidative stress, circadian rhythms, and opioids has been shown in Figure 8.

## 14 FACTORS INFLUENCING THE INTERACTION BETWEEN CIRCADIAN RHYTHM AND OPIOIDS

### 14.1 Opioid drug–drug interaction

Circadian rhythm has been shown to affect the outcomes of interactions between opioids and other drugs. In this regard, circadian differences were demonstrated for opioid interactions with acute lithium, captopril, calcium channel blockers, and L‐N(G)‐nitroarginine methyl ester (L‐NAME) treatments.^238^, ^239^, ^240^ For example, co‐treatment with L‐NAME, a nitric oxide (NO) synthase (NOS; which catalyzes NO formation from the amino acid L‐arginine) inhibitor, was found to enhance the analgesic impact of morphine at all injection times, and considerably in the late dark period in mice.^29^ In another study, acute treatment with lithium potentiated morphine‐induced analgesia at 9 hours after lights on in mice.^240^

L‐NAME, by suppressing NOS, prevents guanylate cyclase induction to activate the cGMP signaling pathway, which has been shown to critically contribute to the processing of pain in the spinal cord.^241^ Moreover, morphine, by acting on opioid receptors, suppresses adenylate cyclase and thus cAMP production.^242^ Furthermore, notable evidence indicates that NMDA receptor activation is involved in thermal hyperalgesia in the spinal cord, and this effect has been suggested to be in part via the production of NO.^243^, ^244^ Hence, a combination of L‐NAME and morphine treatment, through reducing the activity of both the cGMP and cAMP signaling pathways, may be beneficial in relieving pain.

**FIGURE 9:**
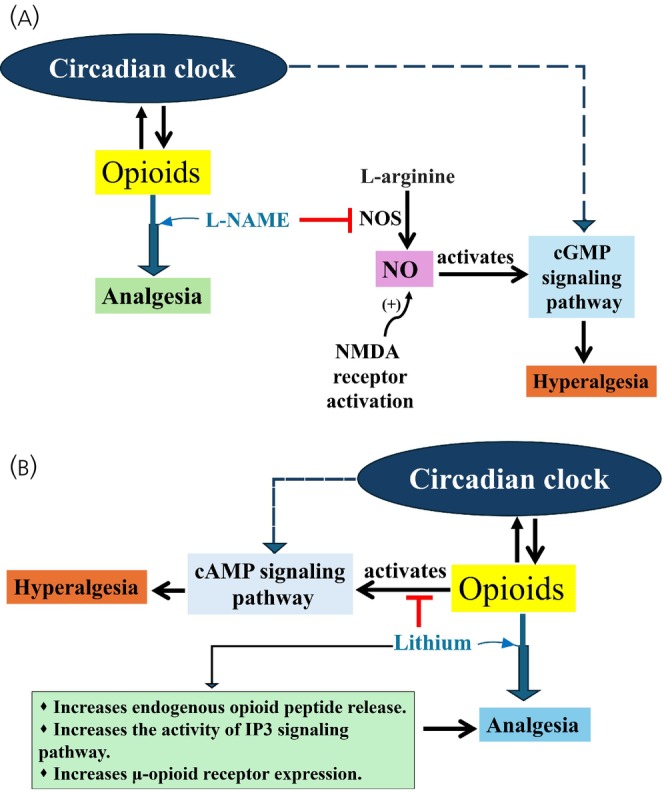
Opioid drug–drug interaction and circadian role. Some medications, including L‐N(G)‐nitroarginine methyl ester (L‐NAME) (Figure 9A) and lithium (Figure 9B) can enhance the pain‐relieving properties of opioids when combined. This can lead to stronger analgesia and potentially lower the need for high opioid doses. L‐NAME acts by inhibiting nitric oxide synthase (NOS), leading to reduced cGMP signaling pathway, while lithium serves by reducing cAMP signaling pathway and increasing the activity of IP3 signaling pathway. The cGMP and cAMP pathways are regulated by the suprachiasmatic nucleus (SCN). The dotted arrow indicates indirect effects.

Multiple mechanisms have been suggested to underlie lithium's effect on the analgesic response of morphine, including enhancing the release of endogenous opioid peptides (beta‐endorphin, Met‐enkephalin, and dynorphin),^245^, ^246^ increasing the activity of the inositol‐1,4,5‐trisphosphate (IP3) signaling pathway, ^247^, ^248^ reducing the level of cAMP produced by opioids,^249^ and increasing μ‐opioid receptor expression in the forebrain.^250^ Rodent studies have revealed that both the guanylate cyclase‐cGMP^251^, ^252^ and adenylate cyclase‐cAMP^253^, ^254^, ^255^ display circadian variations. These circadian fluctuations, as well as the diurnal rhythm in the expression of opioid receptors^256^, ^257^ may be the cause of the observed rhythm in the effect of L‐NAME and lithium. These data suggest that a combination of opioid treatment with certain drugs may be useful in attenuating pain, but the time‐dependent variations in drug–drug interactions should be taken into account. Opioid drug–drug interaction has been shown in Figure 9.

### 14.2 Effect of sex differences

There are conflicting results on the effect of sex on opioid behaviors.^258^, ^259^, ^260^, ^261^, ^262^, ^263^, ^264^, ^265^, ^266^, ^267^, ^268^, ^269^, ^270^ Rodent studies revealed sex differences in opioid analgesia, with a higher and prolonged impact in males than females^259^, ^260^, ^261^; however, these findings are not confirmed by other experiments.^262^, ^263^ Inconsistent findings were also shown in opioid tolerance between males and females,^264^, ^265^, ^266^, ^267^, ^268^, ^269^ with some studies indicating a higher opioid tolerance in males than females^264^, ^270^ and that morphine tolerance developed faster in females than males.^265^, ^266^ Additionally, there are reports indicating more morphine dependency in male rats than females.^264^, ^270^

There is evidence that suggests the presence of an interaction between sex, opioid‐mediated behaviors, and circadian rhythms. For example, sex differences have been reported to affect the daily rhythmicity of morphine consumption in morphine‐treated patients after major abdominal surgery, with greater morphine consumption during the night in males than in females.^271^ In addition, the circadian protein neuronal PAS domain protein 2 (NPAS2), which plays a role in the regulation of circadian‐dependent gene transcription in brain regions involved in opioids and pain modulation, was found to regulate fentanyl‐induced tolerance, hyperalgesia, and dependence in a sex‐specific manner in mice, with higher opioid tolerance and dependency in female NPAS2−/− mice than in male NPAS2−/− mice.^258^ These sexually dimorphic consequences in opioid‐induced tolerance, dependence, and withdrawal could be explained by levels of circulating gonadal hormones. Because opioid tolerance and dependence behaviors were found to be affected by estrogen signaling,^272^ which plays a crucial role in regulating circadian rhythm orchestration of gene expression in the SCN.^273^ Additionally, sex differences have been reported in rhythmic glucocorticoid levels in rodents, with significantly higher peak levels of the glucocorticoid rhythm in females than in males.^274^, ^275^ The rhythmic secretion of glucocorticoids has been reported to induce rhythmic variations in the expression of μ‐opioid receptor in the brainstem and may result in circadian variations in the pain threshold.^210^ The female‐specific enhancement in rhythmic glucocorticoid levels is thought to be due to the activation of an adrenal opioid receptor CXCR7 (a β‐arrestin‐biased G‐protein‐coupled receptor) that is particularly expressed in the female adrenal cortex, but not adequately in males.^275^

### 14.3 Effect of genetic

Epidemiological studies have demonstrated that sensitivity to opioids can predict an individual's susceptibility to opioid abuse and/or addiction,^276^ suggesting that opioid sensitivity and abuse can share a genetic basis. In this regard, a polymorphism in CSNK1E (the gene encoding casein kinase 1‐epsilon) was associated with heroin addiction.^277^ CSNK1E was also shown to increase sensitivity to fentanyl (a potent synthetic opioid).^278^ CSNK1E regulates circadian rhythms^277^, ^278^, ^279^ and its inhibition has been shown to induce phase delays in circadian rhythms in rats.^279^ These data suggest that CSNK1E inhibitors may prevent opioid addiction via circadian clock stabilization.

Furthermore, a relationship between genetic variants in several circadian rhythm‐related genes (VIPR2, PER2, CSNK1E, and AUTS2) was shown in subjects with heroin or methadone addiction.^280^

### 14.4 Effect of age

A significant decline in the antinociceptive effect of opioids (morphine and fentanyl) has been shown in aged mice and rats.^226^ Aging was also found to influence the interplay between opioid effects and circadian rhythms. For instance, increased dark phase morphine‐analgesic effect was reduced in older mice than in younger mice.^31^ Moreover, mice lacking the circadian transcription factor BMAL1 exhibited an early aging phenotype,^236^ which, in turn, may increase the likelihood and severity of opioid adverse effects. In another study, the expression of BMAL1 in several brain regions related to memory, such as hippocampus, was reduced in old hamsters.^281^ These data suggest that perturbed BMAL1 may contribute to aging‐related diseases, including cognitive deficits. Opioid‐related cognitive deficits^282^ may be partly due to a disrupted and dampened BMAL1.

These differences in age‐related effects of opioids and circadian rhythms seem to be at least partly due to abnormalities in oxidative‐stress‐related pathways (Nrf2 pathway and NFκB pathway), leading to the accumulation of ROS.^235^, ^236^, ^283^ In addition, several neurotransmitters and other hormonal substances were demonstrated to decrease with age. For example, a decline in dopamine levels and activity as well as in NE and serotonin levels has been shown in the brain.^284^, ^285^ Furthermore, met‐enkephalin content was reduced in SCN while leu‐enkephalin content was decreased in SCN and PVN of the hypothalamus of old rats.^286^ These reductions in turn may influence the interaction between circadian and opioid systems.

The relationship between sex differences/genetic/age, circadian rhythm, and opioids is summarized in Table 4.

**TABLE 4:** Summary of a relationship between sex differences, circadian rhythm, and opioids. NPAS2: circadian protein neuronal PAS domain protein 2.

Category	Opioid/Circadian
Sex differences	Opioid analgesia: A higher and prolonged effect in males than females (some studies). Opioid tolerance: A higher morphine tolerance in males than females. A higher fentanyl tolerance in female NPAS2−/− mice than male NPAS2−/− mice. Faster development of morphine tolerance in females than males. Opioid dependency: More morphine dependency in male rats than females Higher fentanyl dependency in female NPAS2−/− mice than male NPAS2−/− mice. Sex/opioid/ circadian rhythms: Greater morphine consumption during the night in males than females post‐surgery. Circadian pattern: Higher peak level of the glucocorticoid rhythm in females than males.
Genetic	Polymorphism in CSNK1E gene: Is observed in heroin addiction Increases sensitivity to fentanyl Regulates circadian rhythms Genetic variants in the circadian rhythm‐related genes VIPR2, PER2, CSNK1E, and AUTS2: Are observed in subjects with heroin or methadone addiction.
Age	A reduction in antinociceptive effect morphine and fentanyl in aged mice and rats. A reduction in increased, dark phase morphine‐analgesic effect in older mice than younger mice. Emergence of a premature aging phenotype in mice lacking the circadian transcription factor BMAL1. A reduction in BMAL1 expression in several brain regions in old hamsters.

## 15 CONCLUSIONS

In our modern society, circadian rhythms are increasingly being disrupted due to jet lag, night work, internet/smartphone use, and bright light exposure at night. Studies have demonstrated a disruptive interaction between the circadian rhythms and opioids. Opioids can disrupt circadian rhythms, and disrupted circadian rhythms alter opioid processing pathways. This in turn could diminish the effectiveness of opioid therapy and may even contribute to addictive behavior and the appearance of opioid use disorders. Thus, treatments resynchronizing circadian rhythms may improve addiction behavior and eliminate opioid use disorders. These treatments could target the HPA axis, redox state, and several neurotransmitter systems including the serotonergic, noradrenergic, and dopaminergic systems. Future research can begin to consider one or more systems as potential therapeutic targets to mediate drug response in opioid addiction and explore how these systems may influence individual vulnerability to addiction.

Additionally, evidence of the interactions between sex differences and the circadian system should not be ignored, since some studies have reported the existence of sex‐based differences in daily rhythmicity of the opioid system components as well as in the regulation of chronic opioid‐induced tolerance, hyperalgesia, and dependence. These data suggest that circadian effects on opioid adverse effects may act via distinct pathways that are sex‐specific. Thus, treatments for opioid adverse effects may benefit from sex‐specific pharmacological interventions and behavioral approaches.

Also, combining opioids with certain drugs could potentiate the therapeutic outcomes of opioids via reducing the activity of cGMP and cAMP and increasing IP3 signaling pathways. This in turn allows the use of lower opioid doses, which can decrease the risk of overdose and other adverse side effects of opioids.

A limitation of this study is that some studies included in this review article are the lack of direct evidence evaluating interactions between changes in circadian rhythms, opioid‐related negative effects, and some of the factors mentioned above such as oxidative stress as well as dopaminergic and serotonergic neurotransmission. Thus, further research is needed to validate the preliminary findings acquired from these studies based on direct evidence. Future studies should also explore the exact role of the circadian system in the effects of genetic variations, sex differences, and age on opioid sensitivity.

Together, multiple factors including melatonin, oxidative stress, stress, dopamine, and serotonin might be implicated in circadian‐mediated changes in chronic opioid‐induced adverse impacts. Thus, circadian medicine should be equally regarded in conjunction with therapeutic interventions for chronic opioid‐mediated negative effects. Considering these interconnected relationships may be particularly beneficial for cancer pain management, where opioids are used as the first line of treatment for moderate to severe chronic cancer pain.^287^

## AUTHOR CONTRIBUTIONS

**Nasrin Mehranfard:** Investigation; writing – original draft; methodology; validation; visualization; writing – review and editing; software. **Maedeh Ghasemi:** Investigation; writing – original draft; methodology; writing – review and editing; validation. **Ehsan Saboory:** Conceptualization; investigation; writing – original draft; methodology; validation; visualization; writing – review and editing.

## CONFLICT OF INTEREST STATEMENT

There is nothing to declare.

## PEER REVIEW

The peer review history for this article is available at https://www.webofscience.com/api/gateway/wos/peer‐review/10.1111/jne.70065.

## Data Availability

The data that support the findings of this study are available on request from the corresponding author. The data are not publicly available due to privacy or ethical restrictions.
